# Sudden Infant Death Syndrome and the Genetics of Inflammation

**DOI:** 10.3389/fimmu.2015.00063

**Published:** 2015-02-20

**Authors:** Linda Ferrante, Siri Hauge Opdal

**Affiliations:** ^1^Department of Research in Forensic Pathology, Norwegian Institute of Public Health, Oslo, Norway; ^2^Department of Pathology, Oslo University Hospital, Oslo, Norway

**Keywords:** genetics, immune system, interleukins, infection, SIDS

## Abstract

Several studies report signs of slight infection prior to death in cases of sudden infant death syndrome (SIDS). Based on this, a hypothesis of an altered immunological homeostasis has been postulated. The cytokines are important cellular mediators that are crucial for infant health by regulating cell activity during the inflammatory process. The pro-inflammatory cytokines favor inflammation; the most important of these are IL-1α, IL-1β, IL-6, IL-8, IL-12, IL-18, TNF-α, and IFN-γ. These cytokines are controlled by the anti-inflammatory cytokines. This is accomplished by reducing the pro-inflammatory cytokine production, and thus counteracts their biological effect. The major anti-inflammatory cytokines are interleukin-1 receptor antagonist (IL-1ra), IL-4, IL-10, IL-11, and IL-13. The last decade there has been focused on genetic studies within genes that are important for the immune system, for SIDS with a special interest of the genes encoding the cytokines. This is because the cytokine genes are considered to be the genes most likely to explain the vulnerability to infection, and several studies have investigated these genes in an attempt to uncover associations between SIDS and different genetic variants. So far, the genes encoding IL-1, IL-6, IL-10, and TNF-α are the most investigated within SIDS research, and several studies indicate associations between specific variants of these genes and SIDS. Taken together, this may indicate that in at least a subset of SIDS predisposing genetic variants of the immune genes are involved. However, the immune system and the cytokine network are complex, and more studies are needed in order to better understand the interplay between different genetic variations and how this may contribute to an unfavorable immunological response.

## Introduction

**Figure 1:**
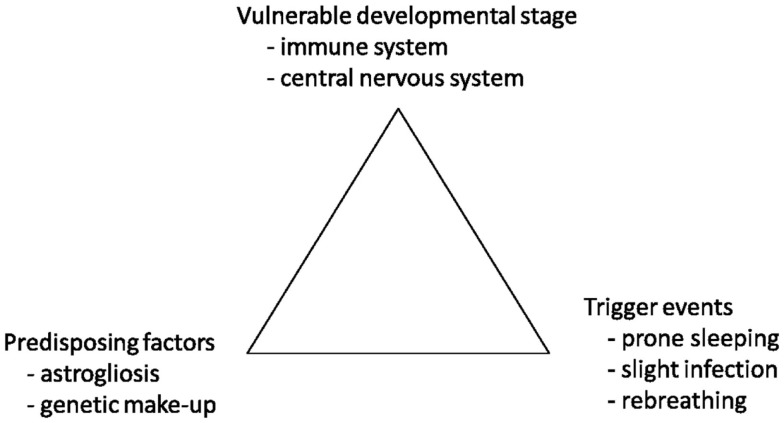
**The hypothesis of a fatal triangle in SIDS**. Modified with permission from Rognum and Saugstad (2).

Sudden infant death syndrome (SIDS) is defined as the sudden unexpected death of an infant <1 year of age, with onset of the fatal episode apparently occurring during sleep, that remains unexplained after a thorough investigation, including performance of a complete autopsy and review of the circumstances of death and the clinical history (1). Even though there have been numerous studies trying to understand the pathophysiological mechanisms of SIDS, we still not fully understand what causes these deaths, or how to prevent them. The fatal triangle developed by Rognum et al. (2) suggest that SIDS occur when an infant at the same time is in a vulnerable developmental stage, with a rapid development of both the central nervous system (CNS) and the immune system, has predisposing factors such as an unfortunate genetic “make-up” or brainstem astrogliosis, and that trigger events such as slight infection, prone sleeping, maternal smoking, or overheating are present (Figure 1) (2).

## SIDS and Infection

There is compelling evidence for a dysfunctional immune response in SIDS, and already in 1947, Werne et al. suggested that respiratory infection was the cause of death in an otherwise healthy infant (3). Since then, there have been numerous reports and papers describing signs of slight infection in SIDS infants (4–7). An immunological “overreaction” has been postulated since about half of the SIDS victims have had symptoms of slight infection in the days before death (8, 9).

Arnon et al. have hypothesized that some infants might suffer respiratory arrest due to botulinum toxin produced by *Clostridium botulinum* (10). From a cohort of 280 infants, they showed that botulinum toxin was present in 10 infants, of which 9 had been diagnosed as SIDS (10). Stoltenberg et al. have reported immune stimulation in both the upper airways and intestines, showing that SIDS had higher number of IgM immunocytes in the tracheal wall than controls, but significantly lower numbers of IgA and IgM immunocytes than cases of infectious death (11). In the duodenal mucosa, the number of IgA immunocytes was higher in SIDS cases than in controls. These findings indicate that the mucosal immune system is activated in a large proportion of SIDS (11). It is also shown that SIDS has higher IgG and IgA immunocyte density in the palatine tonsillar compartments than controls (12). Furthermore, salivary glands have a higher number of CD45+ stromal leukocytes, as well as intensified epithelial expression of HLA-DR and secretory component, and increased endothelial expression of HLA class I and II (13). These observations confirm that the immune system is activated in SIDS, probably with release of certain cytokines that are known to up-regulate epithelial expression of HLA-DR and secretory component (13).

A real breakthrough for the immunological overreaction theory was the demonstration by Vege et al. (8, 14), who showed that SIDS victims who have had signs of slight infection prior to death had both increased number of IgA immunocytes and HLA-DR expression in their laryngeal mucosa, as well as increased levels of IL-6 in their cerebrospinal fluid (CSF). In fact, half of the SIDS victims had CSF IL-6 concentrations in the same range as victims of meningitis and septicemia (8). A further support for the infection theory is a study performed on registry data from Norway and Sweden, which suggests that there is a co-variation between epidemics of whooping cough and SIDS (15). The association was stronger in Sweden than in Norway, which may reflect that Swedish infants are not vaccinated against *Bordetella pertussis* while the Norwegians infants are (15).

Stray-Pedersen et al. showed that SIDS victims with positive *Helicobacter pylori* stool antigen (HpSA) immunoassay had elevated IL-6 in the CSF compared to SIDS victims with negative HpSA test (16). Furthermore, detection of *Helicobacter pylori* antigen in stool was found associated with SIDS and death due to infection, indicating that this bacteria may represent a contributing factor to sudden death during the first months of life (16).

Surfactant protein A (SP-A) is a protein produced in the lungs, with a major purpose to reduce the surface tension at the alveolar air–liquid interface. Furthermore, it takes part in regulation of the inflammatory process. Interestingly, with regard to SIDS, there is a drop in alveolar SP-A expression in the first months after birth (17), corresponding to the classical age peak of SIDS. Thus, it may be hypothesized that this transient low expression of SP-A may be a part of the increased vulnerability for SIDS at that age (17).

It is also suggested that *Staphylococcus aureus* (*S. aureus*) are involved in events leading to SIDS (18). Based on observations from samples collected from the intestinal tract in SIDS compared with samples from feces from a group of healthy controls, it was shown that *S. aureus* and staphylococcal enterotoxins were more prevalent in SIDS. However, as much as 40% of the controls were positive for *S. aureus*, indicating that this bacteria is common in infants, and that the detection may not be seen as a support for the diagnosis of SIDS (18).

Another study investigated pyrogenic toxins of *S. aureus* in SIDS infants from different countries (19). The study reported these pyrogenic toxins in >50% of SIDS infants from three different countries; Scotland, France, and Australia, and suggest that further investigation into the effect of the toxins may be important (19). A study by Blackwell et al. found that the prevalence of *S. aureus* in nasopharyngeal flora was significantly higher in SIDS cases compared to age-matched healthy controls (20). Furthermore, SIDS found in a prone sleeping position more often had symptoms of slight infection prior to death than the babies put to sleep on their back.

The relationship between laryngeal immune stimulation, clinical signs of slight infection prior to death, and high levels of IL-6 in CSF may indicate an interaction between the immune system and the CNS (14). The assumption of such a relationship is strengthened by the recently reported increased IL-6 receptor expression on serotonergic cells in brain stem nuclei involved in respiratory regulation in SIDS cases compared to controls (21). Almost half of the investigated SIDS cases had signs of mild infection prior to death, and the study provides evidence for aberrant interactions in SIDS infants between IL-6 and the area of the brain stem involved in protective responses to hypercapnia, potentially induced by the combined effect of prone position and mild infection.

## Complement Component C4

The first gene involved in the immune system to be investigated with regard to SIDS was the gene encoding complement component C4. This gene consists of two loci, C4A and C4B, and is highly polymorphic. Partial deletions of the C4 gene are common and found in 5–20% of Caucasians (22). The C4 gene has been investigated in both German and Norwegian SIDS victims, but none of these studies detected any differences between SIDS cases and controls with regard to allele frequencies (23–25). However, two of the studies revealed an association between slight infections prior to death and partial deletions of either the C4A or the C4B gene, which may indicate that this combination represents increased risk of sudden infant death (Table 1) (23, 25).

**Table 1:** **C4 and HLA-DR gene variants investigated in cases of SIDS**.

Gene	Variant	SIDS^a^	Controls^a^	Findings, reference
C4	Deletion of gene	40	47	In SIDS: association between slight infection prior to death and partial C4 deletions (*p* = 0.013) (23)
	Deletion of gene	39	183	No significant findings (24)
	Deletion of gene	104	84	In SIDS: association between slight infection prior to death and partial C4 deletions (*p* = 0.039) (25)
HLA-DR	Allele analyses	40	47	No significant findings (23)
		39	183	HLA-DR2 associated with SIDS (*p* = 0.002) (24)
	Allele analyses	16	181	Underpowered, no significant findings (26)

*^a^Number of cases*.

## HLA-DR

Numerous diseases are associated with different alleles of the major histocompatibility complex, and HLA-DR has been investigated in a few SIDS victims (Table 1). There has been reported a significant decreased frequency of HLA-DR2 in a study including 39 SIDS cases and 183 controls (24). However, Schneider et al. (23) investigated 40 SIDS cases and found no significant difference in the HLA-DR gene frequencies between the SIDS cases and the controls, an observation, which was later confirmed in a study of 16 Norwegian SIDS cases (26).

## IL-10

The genes most likely to explain the vulnerability to infection seen in SIDS are the cytokine genes. Several studies have investigated these genes in an attempt to uncover associations between SIDS and different genetic variants, and one of the most investigated is IL-10.

IL-10 is an important immune regulatory cytokine that downregulates the production of pro-inflammatory cytokines, such as IL-6 and TNF-α. Levels of IL-10 control the balance between inflammatory and humoral responses, and IL-10 therefore plays an important role in the development of infectious disease. Variability in IL-10 production has a hereditary component of approximately 75%, and the SNPs in the promoter region in position −1082, −819, and −592, as well as the microsatellite IL-10R and IL-10G, are collectively responsible for the production of the protein (27, 28).

The first study investigating the IL-10 gene in SIDS was by Summers et al., who investigated 23 SIDS cases and found that SIDS was associated with both the ATA haplotype and the −592A allele (29). However, according to the authors, babies who died of other causes might have been included in the SIDS group, which may have influenced the results (29). A Norwegian study of 214 SIDS cases was unable to confirm the association to SIDS, but found an association between the ATA haplotype and the ATA/ATA genotype and infectious death (Table 2) (30). The latter study also investigated the microsatellites, and found a higher percentage of the genotypes G21/G22 and G21/G23 in cases of infectious death compared to SIDS, and a higher percentage of G21/G22 in the SIDS cases compared to controls, while there were no differences between the groups for the IL-10R area (Table 2) (30). Subsequent studies investigating this gene have reported conflicting results, a small study investigating that 38 SIDS cases found an association between −592A and ATA/ATA and SIDS (31), while other studies did not (Table 2) (32, 33). An Australian study investigated the −1082A/G polymorphism in SIDS cases and SIDS parents from different countries, but did not find any differences in genotype frequency between this combined SIDS population and controls, neither between SIDS parents and controls (Table 2) (34). This study also investigated the influence of genotype and smoking on IL-10 responses. They found that the pooled data from smokers had significantly lower levels of IL-10 responses to TSTT, but there were no significant differences for smokers compared with non-smokers for the three genotypes (34). The lowest levels of IL-10 responses were observed among smokers who were homozygous for the −1082A allele, which is most prevalent among Aboriginal Australians and Bangladeshis. This is interesting, as the major difference between the risk factors for SIDS in these two groups is the level of exposure of infants to maternal smoking.

**Table 2:** **Interleukin and cytokine gene polymorphisms investigated in cases of SIDS**.

Gene	Variant	SIDS^a^	Controls^a^	Findings, reference
IL-10	−1082A/G	214	136	ATA haplotype associated with infectious death (*p* = 0.007), no association to SIDS (30)
	−819C/T	
	−592A/C	
	−1082A/G	38	330	−592A allele associated with SIDS (*p* = 0.004) (31)
	−819C/T	
	−592A/C	
	−1082A/G	23	100	Underpowered, no significant findings (32)
	−819C/T	
	−592A/C	
	−1082A/G	123	406	No significant findings (33)
	−592A/C	
	−1082A/G	85	118	No significant findings (34)
	IL-10G	214	136	G21/G22 associated with SIDS (*p* = 0.005) (30)
	IL-10R	214	136	No significant findings (30)
IL-6	−174G/C	25	136	−174G associated with SIDS (*p* = 0.034) (35)
	−174G/C	96	467	−174GG associated with Australian SIDS (*p* = 0.02) (36)
	−174G/C	175	71	No significant findings (37)
	−572G/C	148	131	No significant findings (38)
IL-1α	+4845G/T	204	131	No significant findings (39)
	VNTR intron 6	204	131	VNTR A1A1/+4845TT combination associated with SIDS (*p* < 0.01) (39)
IL-1Ra	+2018T/C	87	122	No significant findings (40)
	VNTR intron 2	204	131	No significant findings (39)
	VNTR intron 2	113	218	2/2 genotype and A2 allele more common in SIDS (*p* = 0.026 and *p* = 0.004, respectively) (41)
	VNTR intron 2	49	94	A2 allele associated with SIDS (*p* = 0.007) (42)
TNF-α	−308A/G	23	330	Underpowered, no significant findings (29)
	−238 G/A	23	100	Underpowered, no significant findings (32)
	−308G/A	
	−1031T/C	148	131	−238GG associated with SIDS (*p* = 0.022). SNP profiles −1031CT/−238GG/−857CC/−308GG and −1031TT/−238GG/−857CC/−308AA associated with SIDS (*p* = 0.03 and *p* = 0.05, respectively) (43)
	−857C/T	
	−308G/A	
	−244G/A	
	−238G/A	
	−308A/G	89	267	−308AA associated with Australian SIDS (*p* = 0.03) (44)
IL-8	−251A/T	148	131	IL-8 −251AA/AT and IL-8 −781CT/TT more frequent in SIDS found dead prone (*p* = 0.006 for both) (38)
	−781C/T	

*^a^Number of cases*.

*Genes and SNPs with no significant findings are given in Table 3*.

Based on the findings in these studies, one might speculate that in some situations an infant with an unfavorable IL-10 genotype may exhibit aberrant IL-10 production, which in turn leads to a disturbed immunological homeostasis and an increased risk for sudden death. This may be especially unfavorable if exposed to smoking, in particular *in utero* or if a smoking mother is breast-feeding.

## IL-6

IL-6 is an acute phase protein that induces B- and T-cell growth and differentiation. IL-6 is also an important mediator of fever, and influences the effect of other cytokines. The first study of the IL-6 gene in cases of SIDS was a British study that included common polymorphisms in the genes encoding IL-4, IL-6, IFN-γ, TGF, and VEGF (35). They found significant differences for the genes encoding IL-6 and VEGF: the genotypes IL-6 −174GG, and VEGF −1154AA were more frequent in SIDS cases than in controls (Table 2) (35). Even though only a small number of SIDS cases were included, the authors suggest that the causation of SIDS is related to both fetal lung development and an infant’s innate ability to mount an inflammatory response to infection (35). The findings in the IL-6 gene have been confirmed in a study of Australian SIDS cases (36), but not in a Norwegian study (Table 2) (37). In the Australian study, it was in addition found a relationship between IL-6 responses to endotoxin, IL-6 genotype, and smoking status (36). A study by Ferrante et al., investigated the −572G/C polymorphism in the IL-6 gene in 148 SIDS cases, but it did not find any association between this SNP and SIDS (Table 2) (38). A study evaluating the correlation between HLA-DR expression in laryngeal mucosa and interleukin gene variation found that 12 of 13 SIDS cases (92%) with high HLA-DR expression, prone sleeping position, and sighs of infection prior to death had the IL-6 −176 CG/CC genotypes (*p* = 0.01) (45).

## IL-1

IL-1 is a pro-inflammatory cytokine that induces the synthesis of acute phase proteins, and also induces fever. There are two structurally distinct forms of IL-1: IL-1α, which is the acidic form and IL-1β, which is the neutral form. IL-1 is regulated by the competitive antagonist IL-1Ra. The polymorphisms IL-1β −511C/T and IL-1Ra +2018T/C have a significant effect on the IL-1β levels. An Australian study investigated a combined SIDS group with cases from different countries with European controls, but did not find any association between those SNPs and SIDS (Table 2) (40). It was, however, shown that smoking had a significant effect on both IL-1β and IL-1ra responses to endotoxin, and that this effect differed according to genotype (40). This finding is highly interesting, since maternal smoking is one of the most well-known risk factors for SIDS (9, 46, 47).

A Norwegian study investigated a variable number of tandem repeat (VNTR) in intron 6 and the SNP in +4845G/T in the IL-1α gene, as well as the −511C/T polymorphism in the gene encoding IL-1β and a VNTR in intron 2 of the gene encoding IL-1ra (Tables 2 and 3) (39). When investigating each polymorphism separately, no association to SIDS was found. However, when combining VNTR and SNP genotypes, an association between the gene combination IL-1α VNTR A1A1/+4845TT and SIDS was disclosed, 16% of the SIDS cases had this combination compared to 1.8% of the controls (*p* < 0.01) (Table 2). In the SIDS group, it was also found that the genotypes IL-1β −511CC/CT were significantly more frequent in the SIDS victims who found dead in a prone sleeping position compared with SIDS victims who found dead in other sleeping positions (*p* = 0.004) (39).

**Table 3:** **Genes and SNPs with no significant findings with regard to SIDS**.

Gene	Variant	SIDS^a^	Controls^a^	Findings, reference
IL-1β	−511C/T	93	122	(40)
	−511C/T	204	131	(39)
TGF-β	+869C/T	23	330	Underpowered study (29)
	+869T/C	25	136	Underpowered study (35)
IFN-γ	+874A/T	25	136	Underpowered study (35)
	+874A/T	148	131	(38)
	+874A/T	69	221	No association to SIDS, but strong tendency (*p* = 0.06) (48)
IL-4	−590C/T	25	136	Underpowered study (35)

*^a^Number of cases*.

The 89bp VNTR in the IL-1Ra gene have also been investigated in Australian SIDS cases, where it was found that carriage of the 2/2 genotype increased the risk for SIDS compared with the predominant 1/1 genotype (Table 2) (41). Homozygous carriers of allele 2 show a more severe and also prolonged pro-inflammatory immune response compared to other IL-1Ra genotypes (49), which may contribute to the vulnerability to infection seen in SIDS. A smaller study investigating the IL-1ra VNTR in 13 SIDS cases and 103 controls found an association between A2A2 and SIDS (Table 1) (42).

## Tumor Necrosis Factor Alpha

Tumor necrosis factor alpha (TNF-α) is a transmembrane protein produced as a result of the presence of bacterial toxins. TNF-α is an important regulator of immune cells, in addition to stimulate inflammation and controlling viral replication. Two smaller studies have investigated the TNF-α polymorphisms −238A/G and −308A/G, but did not find any association to SIDS (Table 2) (29, 32). Five SNPs in the promoter region of the TNF-α gene have been investigated in a Norwegian SIDS population (43). The study found an association between the genotype −238GG and SIDS (Table 2). In addition, SNP profiles −1031CT/−238GG/−857CC/−308GG and −1031TT/−238GG/−857CC/−308AA found more often in SIDS; this may therefore be unfavorable SNP combinations (43). An Australian study investigated the −308G/A polymorphism in SIDS cases from different countries, and found a significantly higher proportion of the AA genotype among Australian SIDS cases compared to controls (Table 2) (44).

## Other Cytokines

In addition to the genes mentioned above have also SNPs in the genes encoding IL-4 IL-8, IL-12, IL-13, IL-16, IL-18, TGF-β1, and INF-γ been investigated in SIDS (29, 35, 38). A Norwegian study found that the genotypes IL-8 −251AA/AT and IL-8 −781CT/TT were significantly more frequent in SIDS cases found dead in a prone sleeping position compared with SIDS cases found dead in other sleeping positions (Table 2) (38). Further, the IL-8 genotypes −251AA/AT and −781CT/TT were more often observed in SIDS cases with positive HLA-DR and one or more risk factors compared with SIDS cases with negative HLA-DR, no infection, and supine sleeping (45). An Australian study investigating SIDS cases from different countries found a marginal association with the IFN-γ +874AA genotype and SIDS (Table 3) (48).

## Other Immune Genes

An increased vulnerability to infection may also be due to genetic variation in the genes encoding G-proteins. The most investigated polymorphism in the Gβ3 gene is C825T, and it is shown that T-allele results in increased G-protein-mediated signal transduction compared to the C-allele (50). Most interleukin-receptors are G-protein coupled, and an association between the Gβ3 825T allele and increased cell function has been reported (51). A study looking at the C825T polymorphism in SIDS victims, cases of infectious death, and live infant controls, revealed no difference in genotype frequency between SIDS cases and controls (52), but an association between the CC genotype and infectious death was found (*p* = 0.016). This observation may indicate that the presence of the 825T allele exerts a protective effect toward serious infection, perhaps through enhanced G-protein signaling.

Surfactant protein A and surfactant protein D (SP-D) are humoral molecules involved in the innate host defense against various bacterial and viral pathogens. Ten common SNPs that might influence expression of the genes encoding these two surfactants have been investigated in SIDS cases and controls (*p* = 0.08) (53). No difference in genotype distribution was found, even though there was a tendency for the most common SP-A haplotype, 6A2/1A0, to be overrepresented in cases with low immunohistochemical SP-A expression (53). None of the other SP-A haplotypes was associated with high or low SP-A expression, and the same was true for the two investigated SP-D SNPs (53).

Polymorphisms that influence the expression of toxin receptors could contribute to SIDS, at least in the cases where there is evidence of toxin involvement. One gene that influences the expression of receptors for staphylococcal enterotoxin B and C in humans are the TCRBV3S1 gene, and a C–T SNP in a recombination signal sequence (RSS) gene region is shown to influence the expression of the gene (54). This SNP have been investigated in 48 Australian SIDS cases and 96 controls, but no differences were found between SIDS cases and controls (55).

Another protein that might be of importance with regard to endotoxins in SIDS is CD14. The TT genotype of the CD14 −260C/T polymorphism causes a significantly higher density of CD14 receptor expression in monocytes, which makes the individual more sensitive to endotoxin than those with the CC genotype (56). This polymorphism has been investigated in an Australian cohort of 116 SIDS cases and 228 controls (57). No differences were found in genotype frequencies between SIDS cases and controls, and the authors conclude that the CD14 −260C/T polymorphism is unlikely to be involved in SIDS (57).

## Discussion

The many genetic studies within immune genes in SIDS strongly suggest that these infants do have a combination of genotypes making them vulnerable to infections. The predispositions reported so far are mostly common SNPs with association to SIDS, but genetic variants with strong dominance in SIDS still remains to be uncovered. However, the challenges within genetic studies of SIDS are many. First and foremost is the fact that the SIDS diagnosis is an exclusion diagnosis and important challenge, and even though a great effort has been done in order to standardize is still different diagnostic criteria used in different countries. This might result in that infant with other causes of death, such as accidental asphyxia, or interstitial pneumonia might be included in the SIDS group, and thus camouflage a true genetic association.

Another challenge to genetic studies is the genetic variation between different ethnic groups, which makes it difficult to compare results between different studies without including a potential error. This must be taken into account when investigating SIDS. In addition, the investigated SIDS populations are often small, which make the genetic studies more difficult to interpret. The number of SIDS cases a year in, for instance, Norway is at the moment about 20, making it difficult and time consuming to be able to collect a large number of cases.

The fact that the number of cases and controls in each study often is small in relation to the number of statistical tests that is undertaken increases the risk of false negatives. Several studies include <25 SIDS cases, which makes it difficult to draw any firm conclusions. Even so, to our best of knowledge are all studies investigating immune genes in SIDS included in Tables 1–3, in order to give a comprehensive survey of the research done regarding this topic so far. A large scale GWAS study might be an important next step. The drawback of this method is, however, that important information may be lost due to the corrections that have to be made when investigating such a vast number of polymorphisms, in addition to the problem with limited number of cases. Even so, one might find polymorphisms that could be expected to have a small effect on the SIDS risk. This may be important as these gene variants may point directly to the underlying cause, such as for instance a sub-optimal immune reaction.

Despite the limitations, association studies are still able to report several genetic variations within many immune genes that might be associated with SIDS (Tables 1 and 2). This strongly suggest that these genes are of importance and hopefully in the future can provide even better and more accurate understanding of the role of the immune system in SIDS. However, immunology is a complicated biological field, and perhaps the most interesting frontiers in the genetics of immunity arise in the interaction between immune activity and other physiological or developmental processes. A better understanding of how genetic variation might influence the functional effect of the encoded proteins and how this can lead to a fatal outcome is important, and hopefully, with the new possibilities present within genetic research, this can be elucidated. Further would an in-depth analysis of the correlation between genotypes, interleukin response, and risk factors, such as smoking exposure, in different SIDS populations be interesting and might shed light on the involvement of the immune response in these deaths.

## Conclusion

The studies so far suggest that some SIDS infants may have a genetic vulnerability in the regulation of the immune system. An unfortunate combination of polymorphisms in genes involved in the immune system, in particular in the cytokine genes, may lead to an imbalance in the immune response and render the infant unable to cope with an infection. However, the immune system and the cytokine network are complex, and more studies are needed in order to better understand the interplay between different genetic variations and how this may contribute to an unfavorable immunological response. The associations observed so far between different polymorphisms and SIDS and environmental factors for SIDS most likely represent only a small part of genetic patterns that may result in an unfortunate immunological reaction.

## Conflict of Interest Statement

The authors declare that the research was conducted in the absence of any commercial or financial relationships that could be construed as a potential conflict of interest.
